# Nidus localization in osteod osteoma by SPECT skeletal scintigraphy: Aid to diagnosis and surgical approach

**DOI:** 10.4103/0972-3919.63594

**Published:** 2010

**Authors:** Santhi Bhushan Murari, N Sujith, M Ranadheer, P Chandra Sekhar, P Aruna Kumari, VVS Prabhakar Rao

**Affiliations:** Departments of Nuclear Medicine and Orthopedics, Nizam's Institute of Medical Sciences (NIMS), Hyderabad, Andhra Pradesh, India

**Keywords:** MDP, nidus, Osteod osteoma, SPECT

## Abstract

Osteod osteoma, although not a common clinical occurrence, does pose problems both in diagnosis and surgical management. Initial plain radiographic diagnosis is sometimes fraught with the limitation of not being able to differentiate from chronic osteomyelitis and stress fracture. CT-aided localization of the nidus is also often inconclusive. Radionuclide single photon emission computed tomography (SPECT) scintigraphy is highly sensitive in localizing the active nidus and also orients the lesion in a three-dimensional plane well, for effective surgical removal

## INTRODUCTION

Osteoid osteoma, a benign and uncommon lesion encountered in the young, in their first three decades of life, often poses a problem both in its diagnostic and surgical management. The spectrum of presentation ranges from its incidental detection to a localized painful condition with nocturnal exacerbation. Although an initial approach by conventional radiography is sought, with limitation in not being able to localize the nidus, a CT scan and Single Photon Emission Computerized Tomography (SPECT) skeletal scintigraphy are often required to localize the lesion accurately. SPECT scintigraphy by its high sensitivity and specificity not only confirms the nidus, but also aids in facilitating the surgical approach, for effective enucleation of the nidus, achieving a complete cure. Case Reports with diagnostic and therapeutic challenges are discussed.

## CASE REPORTS

### Case 1

**Figure 1:**
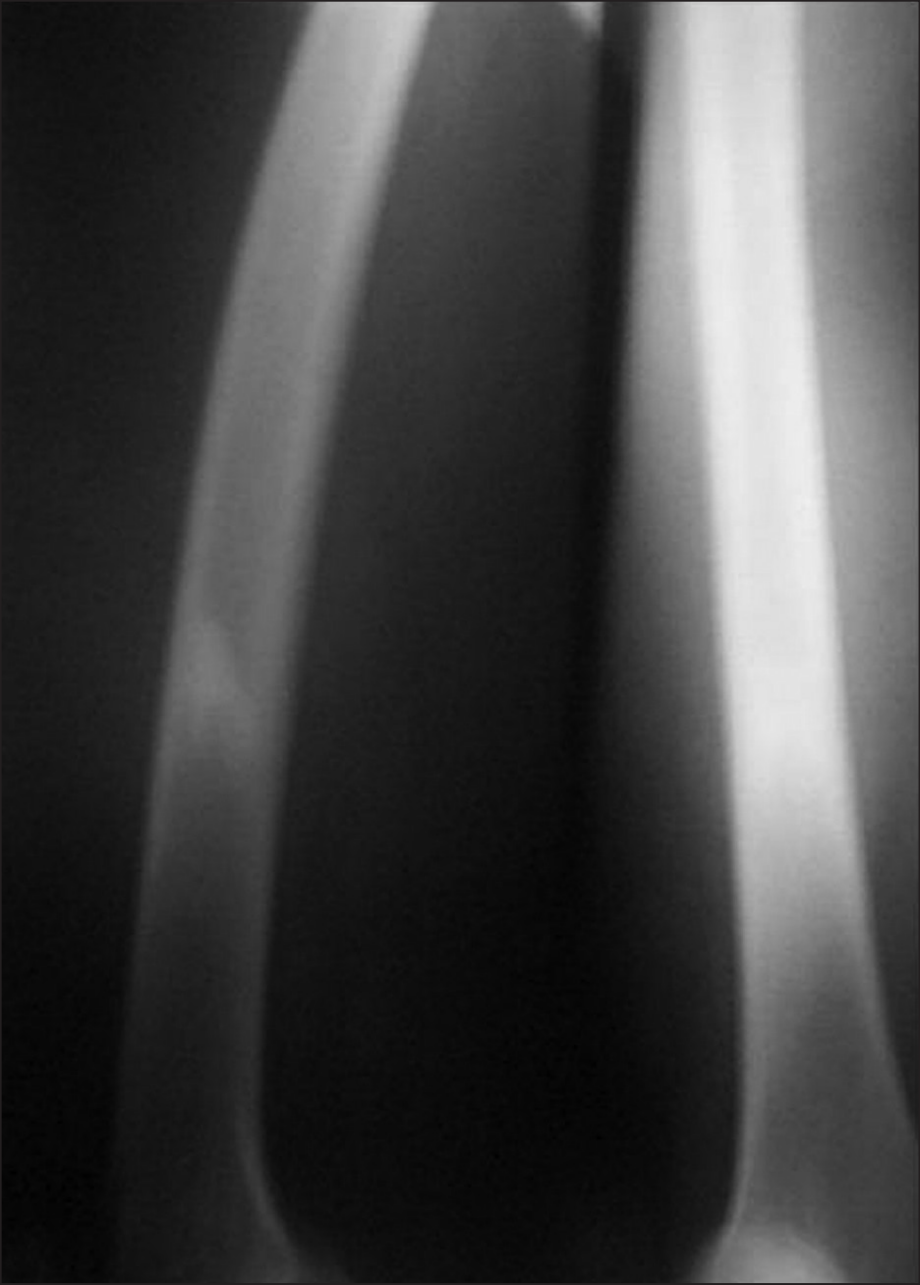
X-ray of the thigh showing only sclerosis

**Figure 2:**
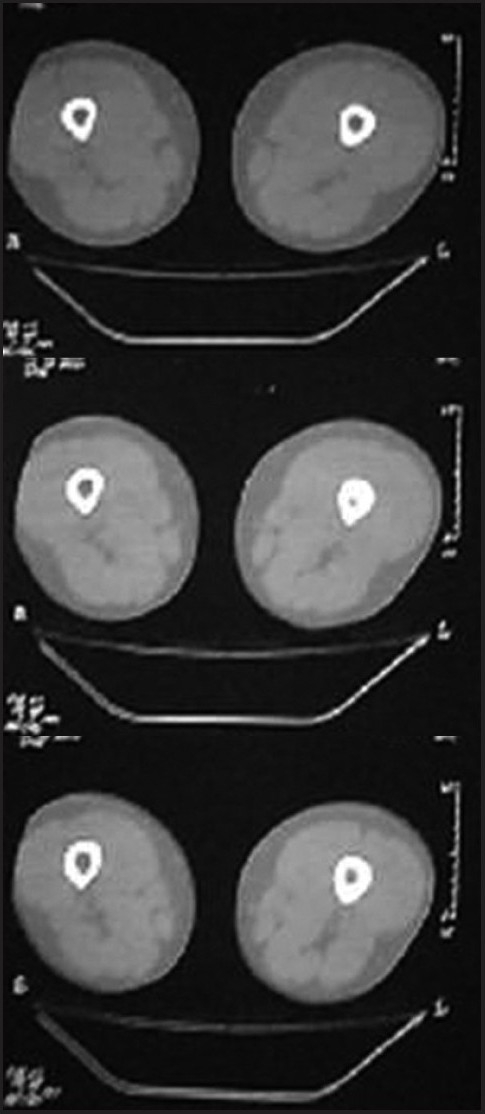
CT of the thigh outlines sclerosis, but not the nidus

**Figure 3:**
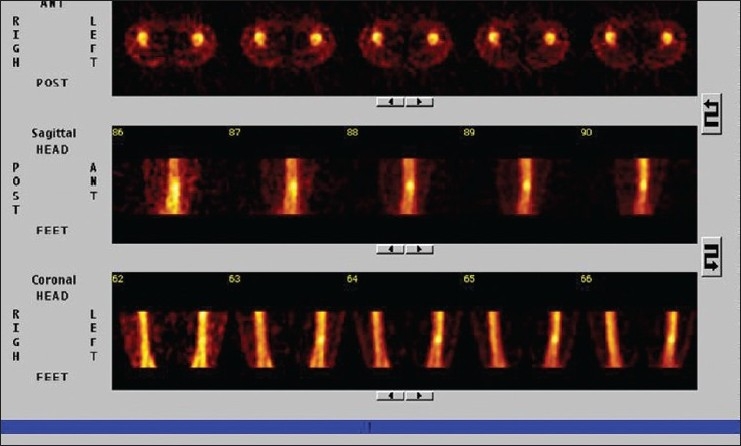
Bone SPECT showing focal uptake of the nidus

**Figure 4:**
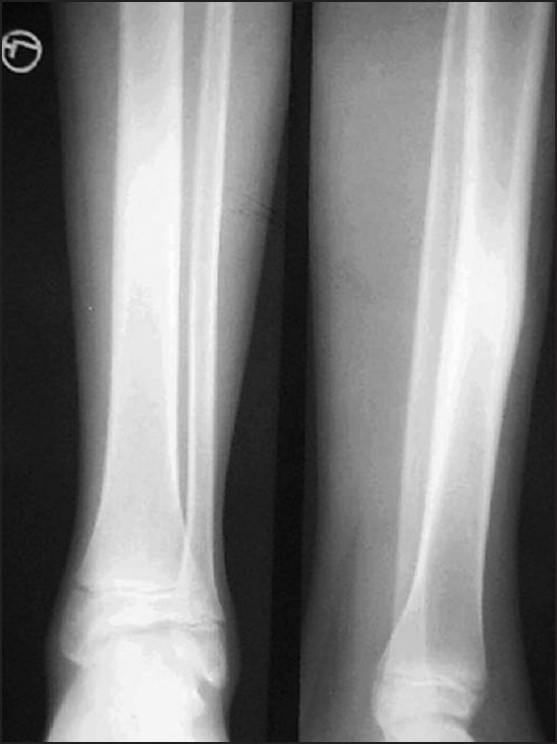
X-Ray of the right leg showing sclerosis on the medial aspect of the right tibia

A 28-year-old male presented with vague pain in the right lower thigh region, of one-month duration. There was no recent history of (H/O) trauma or any constitutional symptoms. Hematological and biochemical parameters were within normal limits. A plain radiograph of the thigh revealed an irregular-shaped, predominantly endosteal sclerosis with no overlying periostial reaction in the lower shaft of the right femur. No obvious nidus was identifiable [Figure 1]. The CT scan of the right lower limb with a bone window could only outline the sclerosis, but not the nidus [Figure 2]. Technetium 99 Metheline Diphosphonate (MDP) with SPECT acquisition revealed a focal avid uptake in the lower shaft of the right femur [Figure 3]. SPECT acquisition revealed the focal avid uptake of the nidus localized to be anterior in the three-dimensional reconstructions of the coronal, sagittal, and axial planes [Figure 4]. Surgical excision was done by an anteriomedial approach and the entire lesion was resected. High Performance Electrophoresis (HPE) confirmed the Osteoid nidus. The patient became symptom-free.

### Case 2

**Figure 5:**
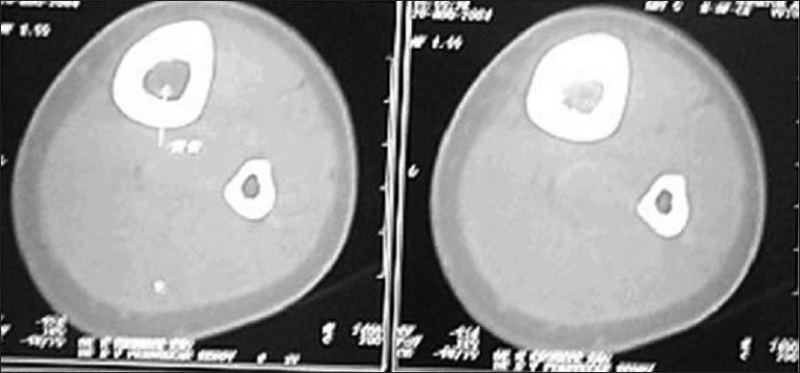
CT of both legs identified sclerosis, but nidus is indefinable

**Figure 6:**
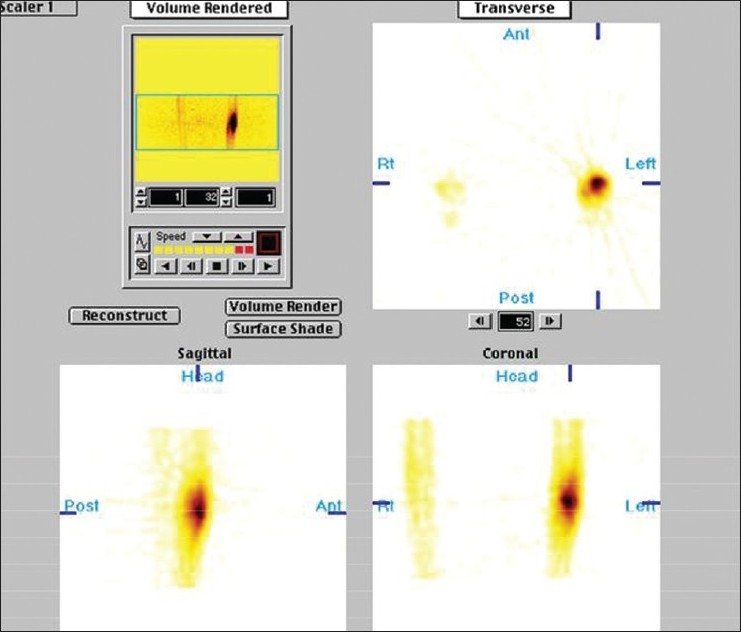
SPECT showing central active nidus with decremental activity toward the periphery, typical of an osteoid osteoma

A 22-year-old male presented with pain in the right leg, of one month's duration. There was a history of trivial trauma in the preceding month. There were no constitutional symptoms such as fever. Local examination revealed boggy swelling on the medial aspect of the right leg with a mild degree of overlying erythema, and mild tenderness. A plain radiograph of the right leg showed a large, diffuse and intense sclerosis on the medial aspect of the right tibia with no periostial reaction [Figure 5]. A CT scan of both legs in the bone window also identified the sclerosis with nidus as being indefinable [Figure 6]. Technetium 99 MDP with SPECT acquisition revealed an active central nidus, concentrating intensely with decremental uptake toward the periphery, typical of osteoid osteoma. The findings also excluded other possibilities such as osteomylitis and stress fracture.

## DISCUSSION

The nomenclature of an osteoid osteoma was introduced by Jaffe in 1935, who defined the lesion as a benign osteoblastic tumor The essential constituent is the NIDUS, which is the core of newly formed osseous tissue and trabeculae in a substrate of highly vascularized osteogenic connective tissue.[1,2] Perifocal sclerosis surrounds the nidus and the degree and intensity of sclerosis is variable Conventional radiography often fails to identify the nidus due to the intense sclerosis masking the radiolucent nidus. A CT scan aids in localizing the nidus by virtue of density analysis using the bone window.[3,4] However, in the midst of the dense sclerosis the hypodense nidus gets masked due to the artial volume effect. The radiological features of a healing stress fracture and osteomylitis, which form the differential diagnosis, often mimic radiological appearances of the osteoid osteoma and make clinical diagnosis and decision-making regarding the surgical approach, difficult.

MDP bone scan is highly sensitive in the diagnosis of osteoid osteoma. A three-phase bone scan shows the initial vascular blush followed by a focal avid tracer uptake in the delayed skeletal phase. The double density sign is the diagnostic feature of the central nidus on a bone scan.[5] The advent of SPECT improves the localization of the nidus in respect to the bony outline where it resides. SPECT skeletal scintigraphy clearly and conclusively localizes the nidus as a focal avid uptake in the middle of the lesion, with a decremental uptake toward the periphery.[6,7] Knowledge of the precise localization of the nidus guides the surgeon to decide on the best surgical approach with minimal intervention and at the same time achieve complete excision of the tumor. Case No. 1 highlights the role played by the SPECT study, not only in confirming the diagnosis of osteoid osteoma by confirming the nidus, but also by spatially localizing it to being more anteriorly located, thus aiding in a precise surgical approach, to contain mutilation and disfigurement. The clinical differential diagnosis of osteoid osteoma includes a healing stress fracture, and osteomyelitis due to the similarity of symptoms, presentation, and overlapping radiological features. Plain radiography and CT find it difficult to localize the nidus due to varying degrees of bony sclerosis masking the central nidus. However, a SPECT study not only confirms osteoid osteoma, but conclusively excludes osteomyelitis and stress fracture by its characteristic features of central avid focus with decremental activity toward the periphery, differentiating it from osteomyelitis and stress fracture, which would appear more diffuse and linearly oriented along the long axis of the bone, as seen in the second case where, in clinical possibility, osteomyelitis or stress fracture were equally possible, but the bone SPECT localized the nidus and confirmed the diagnosis of osteoid osteoma.

## CONCLUSION

Besides confirmation of the diagnosis by identifying the nidus, SPECT scintigraphy also aids the surgical approach by its ability to spatially localize the lesion in a circumferential profile of the bone (anterior, posterior, medial or lateral). Moreover, SPECT localization is useful in bulky and fleshy areas, especially in the thigh region, wherein, Tc99 scintigraphy, by the exact localization of the nidus in a three-dimensional orientation, guides the surgical approach to be more precise, without being more mutilating, and less invasive.
